# Efficacy of epidermal growth factor receptor inhibitors in combination with chemotherapy in advanced non-small cell lung cancer: A meta-analysis of randomized controlled trials

**DOI:** 10.18632/oncotarget.9503

**Published:** 2016-05-20

**Authors:** Minghui Zhang, Hongsheng Guo, Shu Zhao, Yan Wang, Maopeng Yang, Jiawei Yu, Yubo Yan, Yan Wang

**Affiliations:** ^1^ Department of Medical Oncology, Harbin Medical University Cancer Hospital, Harbin, 150081, China; ^2^ Department of Medical Oncology, Tianjin Third Central Hospital, Tianjin, 300170, China; ^3^ Department of Medical Oncology, Heilongjiang Provincial Hospital, Harbin, 150000, China

**Keywords:** non-small cell lung cancer, epidermal growth factor receptor, tyrosine kinase inhibitor, overall survival

## Abstract

The role of a combination of epidermal growth factor receptor tyrosine kinase inhibitors (EGFR-TKIs) and chemotherapy for non-small-cell lung cancer (NSCLC) has not been well established. To clarify this problem, we performed a meta-analysis with 15 studies identified from PubMed, EMBASE and the Cochrane Library. We found that the combined regimen had a significant benefit on progression-free survival (PFS) (hazard ratio (HR) = 0.80; 95% CI = 0.71–0.90; *P* < 0.001) and the objective response rate (ORR) (RR = 1.35; 95% CI = 1.14–1.59; *P* < 0.001). However, the combined regimen had no significant impact on overall survival (OS) (HR = 0.96; 95% CI = 0.90–1.03; *P* = 0.25). Subgroup analysis showed significantly higher OS advantages in EGFR mutation positive patients (*P* = 0.01), never smokers (*P* = 0.01), Asian patients (*P* = 0.02), patients receiving second-line treatment (*P* < 0.001), and those receiving a sequential combination of EGFR-TKIs and chemotherapy (*P* = 0.005). The combination regimen showed a higher incidence of grade 3–4 toxicities (leucopenia, neutropenia, febrile neutropenia, anemia, rash, fatigue and diarrhea). In summary, the combination of EGFR-TKIs plus chemotherapy in advanced NSCLC achieved a significantly longer PFS and a higher ORR but not longer OS. Well-designed prospective studies are needed to confirm these findings.

## INTRODUCTION

Lung cancer is the leading cause of cancer death worldwide [1]. The majority of new cases are advanced non-small-cell lung cancer (NSCLC) at the time of diagnosis, and palliative therapy with platinum-based doublets are the standard therapy [2]. However, no doublet regimen has proven to be superior, and survival outcomes are poor [3]. Therefore, novel agents are urgently needed for this disease, and epidermal growth factor receptor tyrosine kinase inhibitors (EGFR-TKIs) are among the most widely used agents to serve this purpose.

Currently, EGFR-TKIs (gefitinib and erlotinib) are recommended to be the standard treatment option for advanced NSCLC patients harbouring EGFR mutations [4]. These sensitive mutations are found in approximately 10% of Western patients and 63.1% of Chinese patients with NSCLC [5–7]. Several randomized controlled trials that enrolled patients harbouring EGFR-activating mutations demonstrated that EGFR-TKI is superior to chemotherapy in terms of progression-free survival (PFS) and objective response rate (ORR) [8–11]. However, concomitant administration of EGFR-TKIs standard chemotherapy is controversial. The results of previous randomized trials have not shown improved the overall survival among patients with NSCLC [12–23]. However, another trial on the sequential administration of EGFR-TKIs following chemotherapy revealed a significant improvement in overall survival [24]. This could be explained that the sequential administration of EGFR-TKIs following chemotherapy avoided the potential issue of cell cycle–based antagonism between the two regimens. These interesting results are in accordance with several other reports [25–26].

Therefore, we performed a meta-analysis of randomized controlled trials to comprehensively examine the efficacy and safety of EGFR-TKIs in combination with chemotherapy for the treatment of advanced NSCLC and to find the most effective combinatorial strategy.

## RESULTS

### Study selection and characteristics

**Figure 1:**
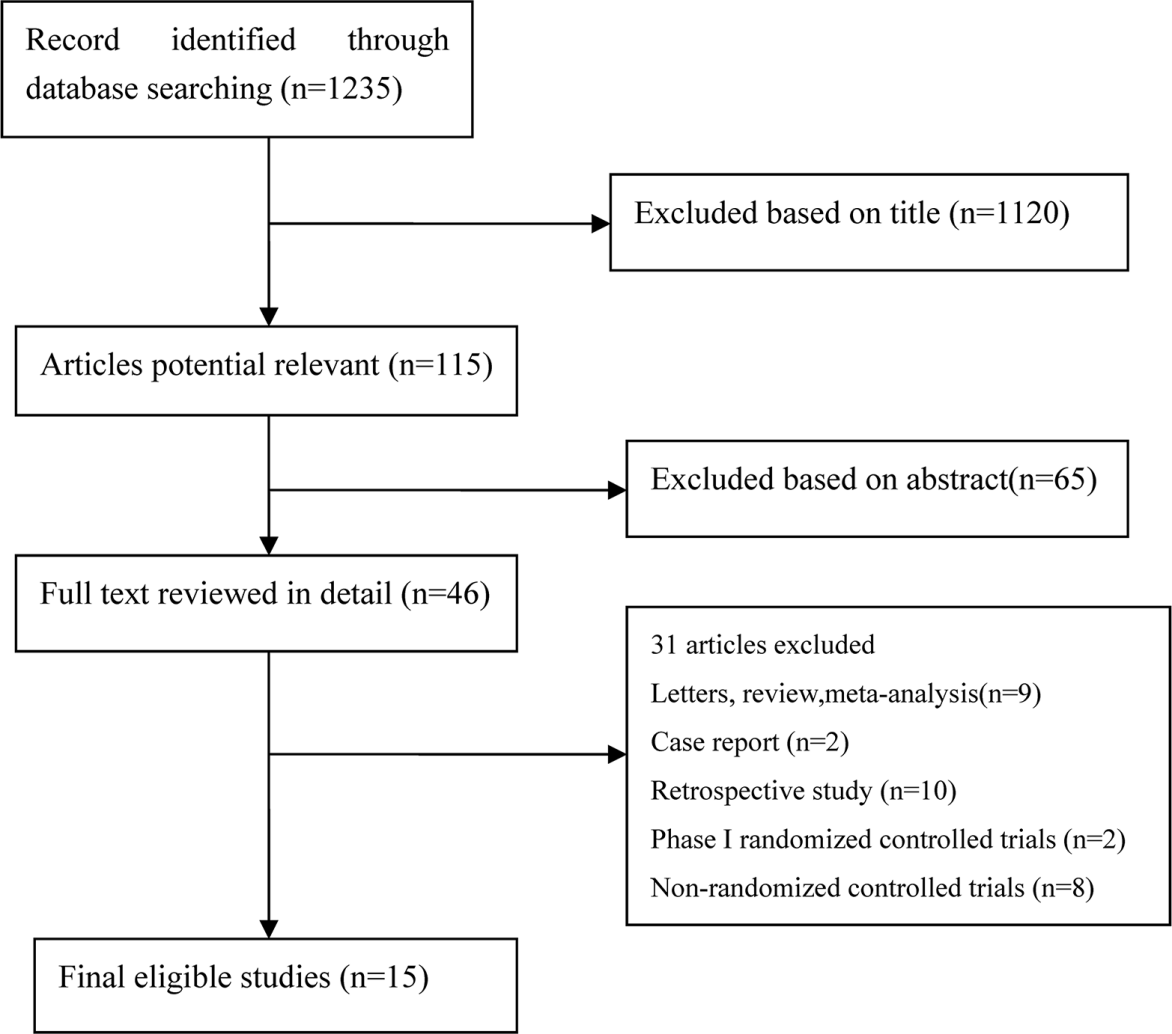
Flow Chart of Study Selection

In the present study, 1,235 articles were identified by the initial search strategy. Through reading the study titles and abstracts, 1,120 articles were removed. After we reviewed the full texts of the 46 potentially eligible articles in detail and identified articles through conference, 15 trials meeting the inclusion criteria were included for the final analysis. A flowchart depicting the study selection is shown in Figure 1. Among these 15 trials, 5,861 patients with advanced NSCLC were investigated. The characteristics of the 15 trials are shown in Table 1.

**Table 1:** Characteristics of the randomized trials included in the meta-analysis

Study	Year	Phase	Line of treatment	Drug delivery	Dominant ethnicity	Treatment comparison	Number of patients	Median age (years)	Female	Never smoker	Activating EGFR-mutant	Jadad score
Aerts	2013	II	Second line	Intercalated	Caucasian	E+DOC or E+PEM	116	62.5	43	9	NA	3
						E	115	64	40	7	NA	
Auliac	2014	II	Second line	Intercalated	Caucasian	E+DOC	75	59.1	14	9	NA	3
						DOC	76	59.7	18	2	NA	
Boutsikou	2013	III	First line	Concurrent	Caucasian	E+DOC+CBP	52	62.5	12	8	NA	3
						DOC+CBP	61	65	4	8	NA	
Dittrich	2014	II	Second line	Concurrent	Caucasian	E+PEM	76	64	30	10	NA	3
						PEM	83	61	34	14	NA	
Gatzemeier	2007	III	First line	Concurrent	Caucasian	E+GEM+DDP	580	60	125	NA	NA	3
						E	579	59.1	142	NA	NA	
Giaccone	2004	III	First line	Concurrent	Caucasian	G+GEM+DDP	365	59	85	NA	NA	4
						G	363	61	101	NA	NA	
Herbst	2004	III	First line	Concurrent	Caucasian	G+TAX+CBP	345	61	146	NA	NA	3
						G	345	63	133	NA	NA	
Herbst	2005	III	First line	Concurrent	Caucasian	E+TAX+DDP	539	62.7	217	72	NA	4
						E	540	62.6	207	44	NA	
Hirsch	2011	II	First line	Intercalated	Caucasian	E+TAX+CBP	71		31	21	12	3
						E	72	NA	44	19	10	
Janne	2012	II	First line	Concurrent	Caucasian	E+TAX+CBP	100	60	58	79	33	3
						E	81	58	49	64	33	
Lee	2013	II	Second line	Intercalated	Asian	E+PEM	78	55.8	58	78	NA	3
						E or PEM	162	54.9	99	162	NA	
Mok	2009	II	First line	Intercalated	Asian	E+GEM+DDP or CBP	76	57.5	22	24	2	3
						GEM+DDP or CBP	78	57	24	28	5	
Soria	2015	III	Second line	Concurrent	Asian	G+PEM	133	60	87	88	127	5
						PEM	132	58	84	91	134	
Wu	2013	III	First line	Intercalated	Asian	E+GEM+DDP or CBP	226	59	94	112	49	5
						GEM+DDP or CBP	225	57.3	85	107	48	
Yu	2014	II	First line	Intercalated	Asian	G+PEM+DDP	58	55.3	25	29	14	3
						PEM+DDP	59	54.9	34	39	18	

Abbreviations: E: erlotinib; G: gefitinib;,DOC: doctaxel; Pem: pemetrexed; TAX: paclitaxel; Gem: gemcitabine; CBP: carboplatin; DDP: cisplatin; NA: not available.

### Progression-free survival

**Figure 2:**
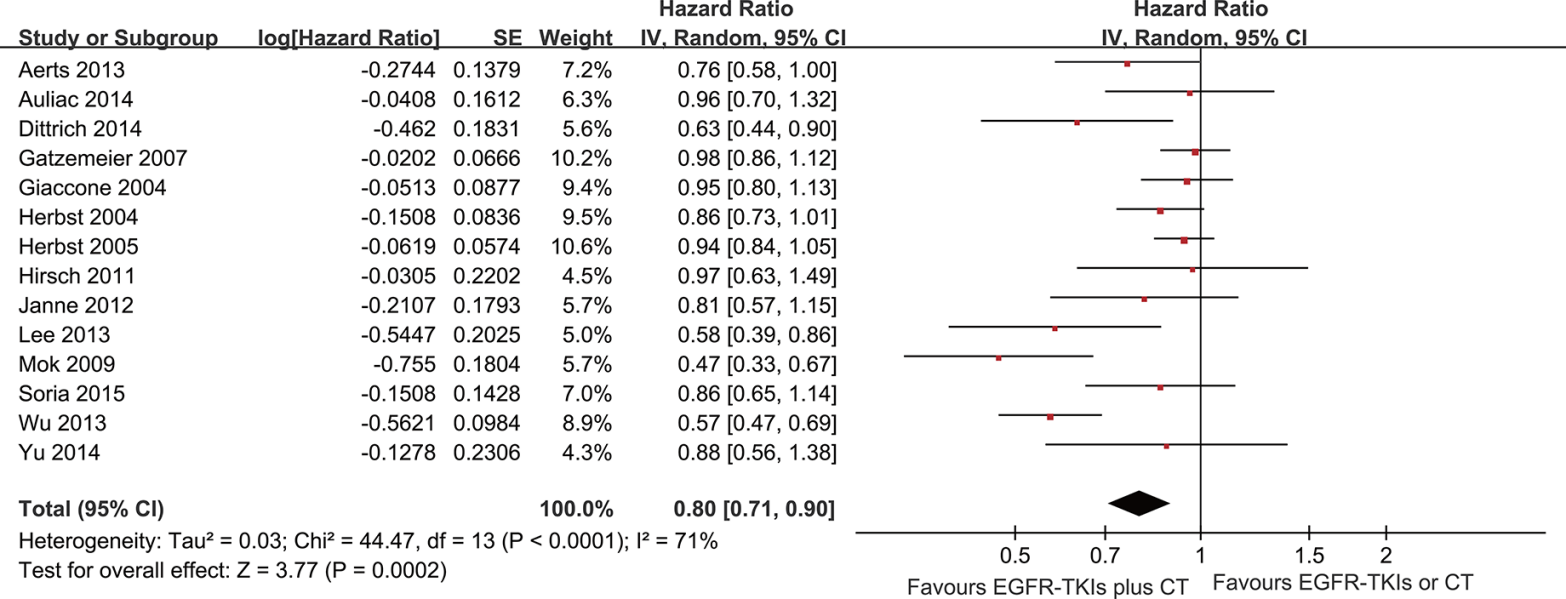
Forest Plot of Meta-analysis for PFS

**Figure 3:**
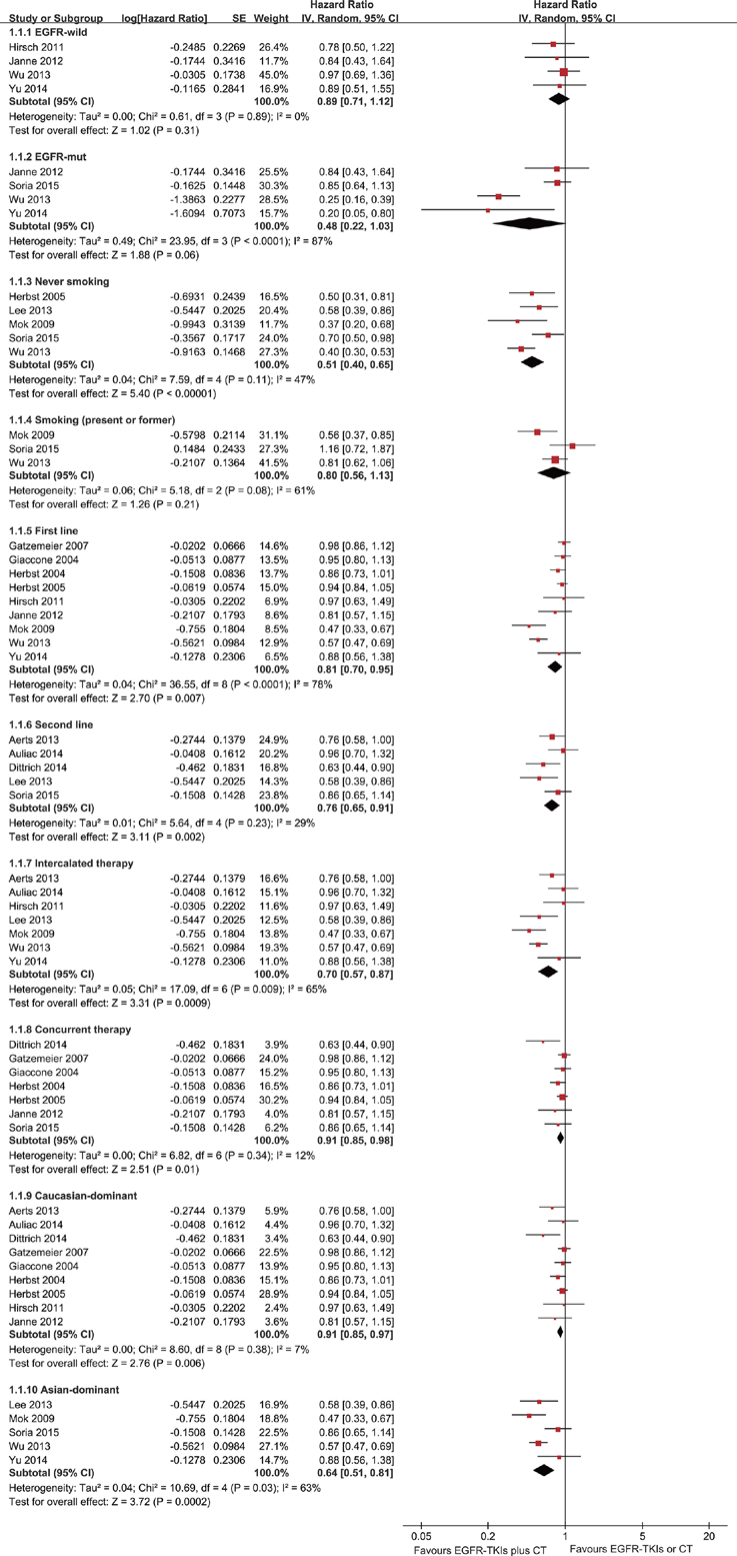
Forest Plot of Subgroup Analysis for PFS

The PFS analysis was based on 14 trials. The meta-analysis showed that the EGFR-TKI combinations significantly reduced the risk of disease progression compared with EGFR-TKIs or chemotherapy alone (HR = 0.80; 95% CI = 0.71–0.9; *P* < 0.001) (Figure 2). Subgroup analysis was conducted according to the EGFR mutation status, smoking status, line of treatment, dose schedules and ethnicity (Figure 3). Subgroup analysis showed that the EGFR-TKI combination was associated with a lower risk of disease progression in never smokers (HR = 0.51; 95% CI = 0.40–0.65; *P* < 0.001). However, EGFR-TKIs did not show a treatment advantage in smoking patients. In addition, the combination group showed a significant improvement in PFS compared to the group receiving chemotherapy alone (HR = 0.76; 95% CI = 0.63–0.91; *P* < 0.002), but this difference was not statistically significant compared to EGFR-TKIs alone (HR = 0.94; 95% CI = 0.86–1.01; *P* = 0.10) (Supplementary Figures S1–S2).

### Overall survival

**Figure 4:**
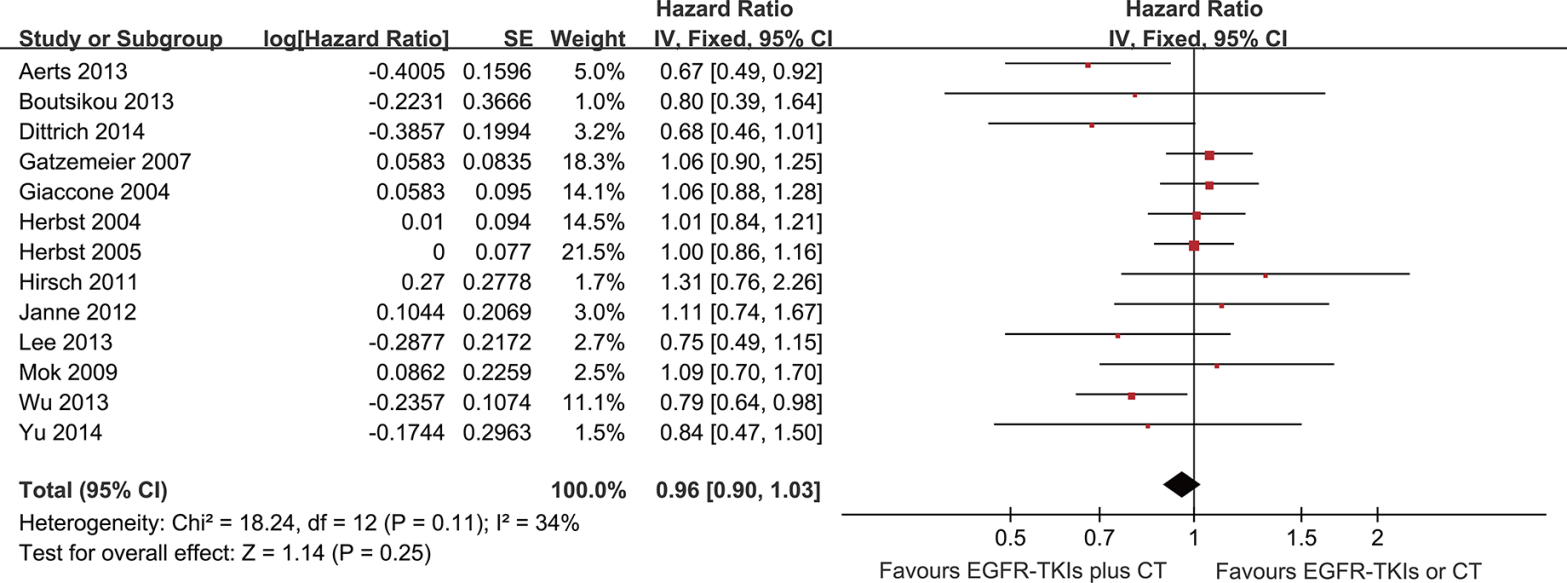
Forest Plot of Meta-analysis for OS

**Figure 5:**
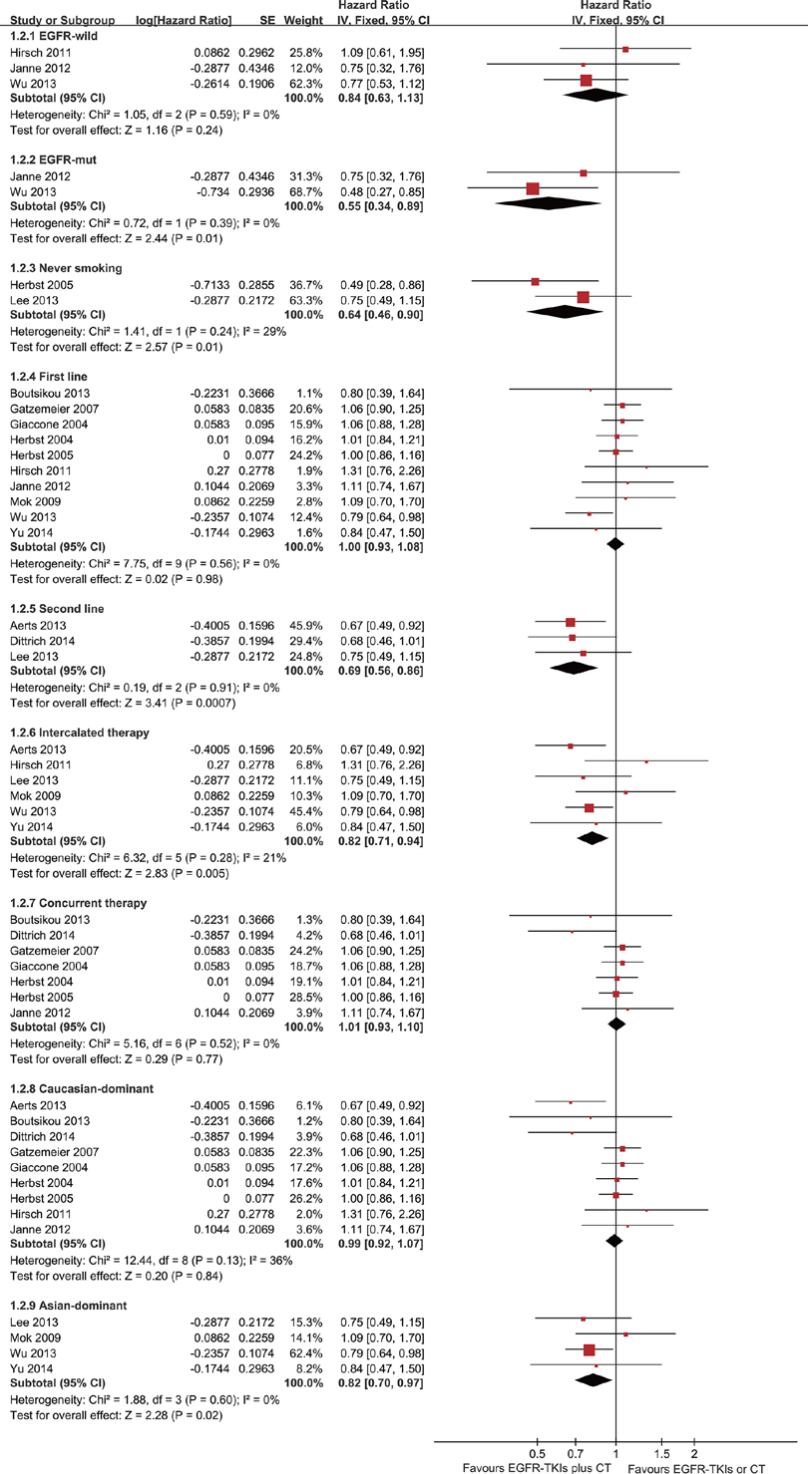
Forest Plot of Subgroup Analysis for OS

Thirteen trials were evaluated for OS. Meta-analysis showed that the EGFR-TKI combination treatment of advanced NSCLC patients did not significantly reduce mortality risk compared with EGFR-TKI or chemotherapy alone (HR = 0.96; 95% CI = 0.90–1.03; *P* = 0.25) (Figure 4). There was no significant heterogeneity in the HR of individual trials (*I*^2^ = 34%; *P* = 0.11). Subgroup analysis demonstrated improvements in patients with EGFR mutations (HR = 0.55; 95% CI = 0.34–0.89; *P* = 0.01) (Figure 5). Furthermore, the patients with advanced NSCLC (mainly the never smokers, patients receiving second-line treatment or intercalated therapy and Asian-dominant groups) would benefit from EGFR-TKI combination therapy. The combination group showed no significant difference in OS compared to the group receiving chemotherapy alone (HR = 0.92; 95% CI = 0.81–1.05; *P* = 0.23) or EGFR-TKIs alone (HR = 0.98; 95% CI = 0.83–1.16.; *P* = 0.83) (Supplementary Figures S3–S4).

### Objective response rate

**Figure 6:**
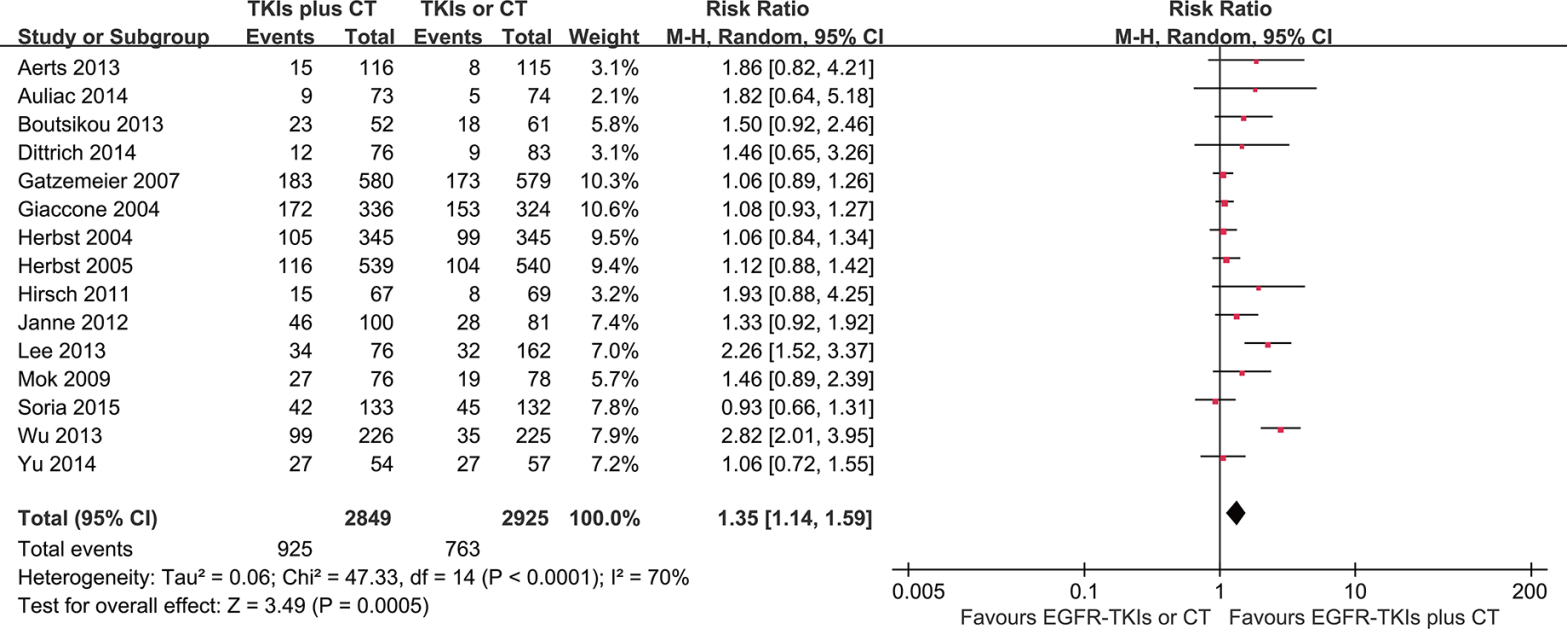
Forest Plot of Meta-analysis for ORR

Data for the objective response rate (ORR) were available from all 15 trials. The results of the collaboration analysis showed heterogeneity among the various studies (*I*^2^ = 71%, *P* < 0.05); thus, random-effects model was employed for the analysis. The meta-analysis demonstrated that the ORR of the EGFR-TKI plus chemotherapy group was significantly higher than the EGFR-TKI- or chemotherapy-alone group (RR = 1.35, 95% CI = 1.14–1.59; *p* < 0.001) as shown in Figure 6.

### Toxicity analysis results

Regarding the incidence of adverse events, compared with the EGFR-TKIs or chemotherapy alone group, the combination group showed a higher incidence of grade 3–4 leucopoenia, neutropenia, febrile neutropenia, anaemia, rash, fatigue and diarrhoea. The complete results are presented in Table 2.

**Table 2:** Grade 3 and higher toxicities between the combined regimen versus chemotherapy or EGFR-TKIs monotherapy

Subgroup	Included trials	Odds Ratio (95% CI)	*P*	Heterogeneity test *I*^2^ (%) *P*	
Hematologic					
Leukopenia	10	1.34 [1.05, 1.72]	0.02	50	0.03
Neutropenia	15	1.47 [1.02, 2.11]	0.04	68	< 0.01
Febrile neutropenia	5	4.95 [2.45, 9.99]	< 0.01	0	0.45
Thrombocytopenia	10	1.25 [1.00, 1.57]	0.05	0	0.44
Anemia	14	1.51 [1.21, 1.89]	< 0.01	0	0.8
Non-hematologic					
Rash	14	3.84 [2.07, 7.14]	< 0.01	58	< 0.01
Anorexia	9	1.65 [0.99, 2.75]	0.06	0	0.57
Fatigue	12	1.53 [1.12, 2.08]	< 0.01	47	0.04
Vomiting	10	1.14 [0.84, 1.54]	0.39	6	0.38
Nausea	10	1.09 [0.79, 1.50]	0.61	0	0.52
Diarrhea	14	3.28 [2.37, 4.54]	< 0.01	3	0.42
Constipation	4	1.00 [0.32, 3.16]	0.99	0	0.54
Dyspnea	6	0.85 [0.60, 1.19]	0.34	0	0.91

### Publication bias

**Figure 7:**
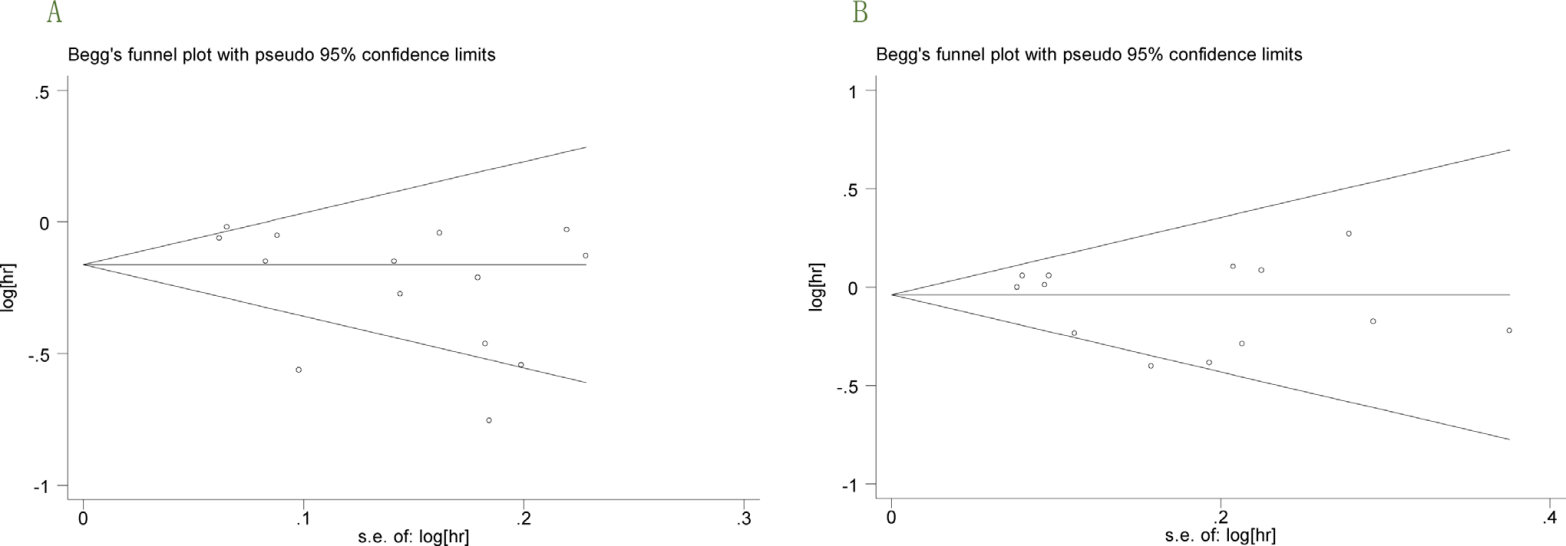
**(A–B)**, (A) Begg's funnel plot with 95 % confidence intervals for PFS publication bias testing. (B) Begg's funnel plot with 95 % confidence intervals for OS publication bias testing.

In the present meta-analysis, no publication bias for PFS and OS was found according to Begg's test (*P* = 0.101 and *P* = 0.583; Figure 7A–7B).

## DISCUSSION

Although platinum-based doublet therapy remains the mainstay of treatment for most patients with advanced NSCLC [27], EGFR-TKIs have assumed an increasingly important role, particularly in patients harbouring EGFR-activating mutations [28]. However, the combination of chemotherapy and EGFR-TKIs has been long debated. To derive a more precise estimate of the effectiveness of EGFR-TKIs in combination with chemotherapy, we systematically reviewed the published studies and carried out a meta-analysis. The meta-analysis demonstrated that the combination of EGFR-TKIs plus chemotherapy in advanced NSCLC achieved significantly longer PFS and higher ORR. The reason may be that the combination regimen enhances anti-proliferative and cytotoxic activities, as demonstrated in human NSCLC cell lines and tumor models [29–30]. However, our results showed that there was no statistically significant difference between the two groups in OS. The reason may be that the differences in OS are potentially affected by the subsequent treatment options. Although the survival data were not statistically significant difference between the two arms, there was a trend in favour of the combination arm.

In the subgroup of patients with EGFR mutations, an improvement in OS was observed for the combination arm. Our results were consistent with the findings of a previous study, which also demonstrated that the addition of EGFR-TKIs to chemotherapy significantly prolonged OS in patients harbouring EGFR mutations [31]. However, no significant difference in OS was noted in EGFR mutation negative patients. These findings demonstrated that the EGFR status may serve as a biomarker to identify patients who can benefit the most from combination therapy and further emphasized the need to test the mutation status at the time of diagnosis. Unfortunately, the EGFR-mutation status was assessed in only a few patients enrolled in eligible trials. Therefore, this result should be interpreted with caution. A head-to-head study is needed to define the value of the combination therapy in the patients with EGFR mutations. Previous studies have suggested that never smokers and Asian patients are more likely to harbour EGFR mutations and benefit more from EGFR-TKIs [32–33]. Furthermore, a history of never smoking was a significant independent predictor for survival in EGFR-TKI treatment [34]. Similarly, this meta-analysis showed that the addition of EGFR-TKIs to chemotherapy had an improvement in the never smoker and Asian-dominant groups.

Two methods of drug delivery were adopted in the combination group, including concurrent and intercalated administration. Previous studies have confirmed a lack of efficacy on the concurrent administration of EGFR-TKIs and chemotherapy [18–19]. In our subgroup analysis, we found that concurrent administration did not confer a survival benefit to patients with advanced NSCLC. Concurrent administration may not be effective because of TKI-induced, G1-phase cell-cycle arrest [35]. Our results are consistent with those in a previous systematic review [36]. The second approach was to administer EGFR-TKIs as intercalated therapy. Our results showed that the intercalated regimen improved the PFS and OS. When EGFR-TKI and chemotherapy are given in a sequentially intercalated way, thus achieving pharmacodynamic separation of the two agents, the inhibitory drug interaction could be avoided [37]. Moreover, EGFR-TKIs were administered not only as a sequential intercalated regimen during chemotherapy but also as maintenance therapy after the end of chemotherapy in intercalated therapy. Subsequent maintenance treatment possibly reduced the effect of the intercalated administration in our pooled analysis because TKI maintenance therapy has been shown to be beneficial in survival [38–39]. Despite this OS benefit in the second-line treatment, only 3 trials were evaluated for OS, and the results should be treated with caution.

In 2013, OuYang et al. [40] also analyzed the treatment effect of the combined regimen on PFS and OS. This meta-analysis was based on only 8 studies. Furthermore, it did not include data from second-line therapy for advanced NSCLC. This study demonstrated that the combined regimen resulted in superior PFS (HR = 0.81, 95% CI 0.69–0.95, *P* = 0.01). The results were consistent with those of our study. Another recent meta-analysis compared chemotherapy plus erlotinib with chemotherapy alone. This also showed an improvement in PFS from the combined regimen, but it did not improve in OS [31]. However, that meta-analysis did not include another important EGFR-TKI, gefitinib, and it did not compare the ORR differences in the two arms.

This meta-analysis had several limitations. First, all of data were extracted from published studies, possibly resulting in publication bias. Second, the EGFR-mutation status was only assessed in a few patients enrolled in eligible trials. Third, all of the clinical trials included in this meta-analysis were conducted in various countries with patients of different ethnicities; these differences may have selected for biases. Fourth, the quality of the included studies may slightly affect the pooled results.

In summary, our study indicated that EGFR-TKIs combined with chemotherapy present a viable therapy for patients with advanced NSCLC. Importantly, the present study suggests that there is a larger magnitude of benefit for Asians, never smokers, and EGFR mutation patients and further suggests that intercalated therapy is the most effective combinatorial strategy.

## MATERIALS AND METHODS

### Search strategy and study selection

Two authors (Zhang MH and Guo HS) independently carried out a comprehensive systematic search for published articles using the PubMed, EMBASE, and Cochrane databases. The deadline of the included articles was September 2015. The search keywords used were as follows: “Erlotinib OR Tarceva OR Gefitinib OR Iressa or EGFR-TKI” and “non-small cell lung OR non-small cell lung carcinoma” and “randomized controlled trial OR controlled clinical trials, randomized”. We also manually reviewed the meeting abstracts of the annual meetings of American Society of Clinical Oncology (ASCO), European Society of Medical Oncology congresses (ESMO) and the World Conference of Lung Cancer (WCLC) from 2004 to 2015. The related references from the included studies were also manually examined.

### Eligibility criteria

The inclusion criteria were as follows: (1) the patients had histopathologically confirmed advanced NSCLC; (2) the combined regimen of EGFR-TKI and chemotherapy was compared with chemotherapy or EGFR-TKI alone; (3) the studies were phase II or III prospective randomized controlled clinical trials; (4) at least one of OS, PFS and ORR was evaluated; (5) the sample size was greater than 50 cases because small samples could introduce marked bias. When duplicate publications were identified, only the newest or most informative single article was selected.

### Data extraction and quality assessment

This meta-analysis was performed in accordance with the Preferred Reporting Items for Systematic Reviews and Meta-Analysis (PRISMA) Statement [41]. Data were extracted independently by two reviewers (Zhang MH and Guo HS), and any disagreements between the two reviewers were resolved by consensus involving a third reviewer (Zhao S). The primary end point of this meta-analysis was OS. The secondary end points included PFS, ORR, as well as grade 3–4 adverse events. For each study, the following information was extracted: author's name, year of publication, phase, line of treatment, drug delivery, dominant ethnicity, treatment comparison, number of patients, median age, number of females, number of smokers, activating EGFR-mutant, ORR and adverse events (grade 3–4 events), hazard ratios (HRs) and 95% confidence intervals (CIs) for PFS and OS. If the HRs were not directly reported, we contacted the authors of the primary studies for additional data. If the author did not respond, we extracted data from survival curves [42]. The quality of the included study was assessed using the Jadad score [43].

### Statistical analysis

Survival analysis was conducted using the intent-to-treat (TTP) population. The risk ratio (RR) was calculated as an effect measure for ORR using the Mantel Haenszel method, and HR was calculated for PFS and OS using the inverse variance method. Statistical heterogeneity was evaluated using chi-squared test and *I*^2^. Statistically significant heterogeneity was defined as a chi-squared *P* value < 0.1 or an *I*^2^ statistic > 50%. If heterogeneity was observed, we used a random-effects model to reduce the impact of heterogeneity on the results. If heterogeneity was not observed, a fixed-effects model was used. The potential publication bias was assessed by Begg's test. All of the statistical analyses were performed using Review Manager Version 5.2 (Revman the Cochrane Collaboration; Oxford, England) and STATA version12.0 (Stata Corporation; College Station, TX, USA). *P* values < 0.05 were considered to indicate statistical significance. All *P* values and 95% CIs were two-sided.

## SUPPLEMENTARY MATERIALS



## References

[1] Siegel RL, Miller KD, Jemal A (2015). Cancer statistics, 2015. CA Cancer J Clin.

[2] Schiller JH, Harrington D, Belani CP, Langer C, Sandler A, Krook J, Zhu J, Johnson DH (2002). Comparison of four chemotherapy regimens for advanced non-small-cell lung cancer. N Engl J Med.

[3] Scagliotti GV, De Marinis F, Rinaldi M, Crino L, Gridelli C, Ricci S, Matano E, Boni C, Marangolo M, Failla G, Altavilla G, Adamo V, Ceribelli A (2002). Phase III randomized trial comparing three platinum-based doublets in advanced non-small-cell lung cancer. J Clin Oncol.

[4] Ettinger DS, Wood DE, Akerley W, Bazhenova LA, Borghaei H, Camidge DR, Cheney RT, Chirieac LR, D'Amico TA, Demmy TL, Dilling TJ, Dobelbower MC, Govindan R (2015). Non-Small Cell Lung Cancer, Version 6. 2015. J Natl Compr Canc Net.

[5] Rosell R, Moran T, Queralt C, Porta R, Cardenal F, Camps C, Majem M, Lopez-Vivanco G, Isla D, Provencio M, Insa A, Massuti B, Gonzalez-Larriba JL (2009). Screening for epidermal growth factor receptor mutations in lung cancer. N Engl J Med.

[6] Paez JG, Janne PA, Lee JC, Tracy S, Greulich H, Gabriel S, Herman P, Kaye FJ, Lindeman N, Boggon TJ, Naoki K, Sasaki H, Fujii Y (2004). EGFR mutations in lung cancer: correlation with clinical response to gefitinib therapy. Science.

[7] Wang R, Zhang Y, Pan Y, Li Y, Hu H, Cai D, Li H, Ye T, Luo X, Zhang Y, Li B, Shen L, Sun Y (2015). Comprehensive investigation of oncogenic driver mutations in Chinese non-small cell lung cancer patients. Oncotarget.

[8] Mok TS, Wu YL, Thongprasert S, Yang CH, Chu DT, Saijo N, Sunpaweravong P, Han B, Margono B, Ichinose Y, Nishiwaki Y, Ohe Y, Yang JJ (2009). Gefitinib or carboplatin-paclitaxel in pulmonary adenocarcinoma. N Engl J Med.

[9] Mitsudomi T, Morita S, Yatabe Y, Negoro S, Okamoto I, Tsurutani J, Seto T, Satouchi M, Tada H, Hirashima T, Asami K, Katakami N, Takada M (2010). Gefitinib versus cisplatin plus docetaxel in patients with non-small-cell lung cancer harbouring mutations of the epidermal growth factor receptor (WJTOG3405): an open label, randomised phase 3 trial. Lancet Oncology.

[10] Rosell R, Carcereny E, Gervais R, Vergnenegre A, Massuti B, Felip E, Palmero R, Garcia-Gomez R, Pallares C, Sanchez JM, Porta R, Cobo M, Garrido P (2012). Erlotinib versus standard chemotherapy as first-line treatment for European patients with advanced EGFR mutation-positive non-small-cell lung cancer (EURTAC): a multicentre, open-label, randomised phase 3 trial. Lancet Oncology.

[11] Maemondo M, Inoue A, Kobayashi K, Sugawara S, Oizumi S, Isobe H, Gemma A, Harada M, Yoshizawa H, Kinoshita I, Fujita Y, Okinaga S, Hirano H (2010). Gefitinib or chemotherapy for non-small-cell lung cancer with mutated EGFR. N Engl J Med.

[12] Auliac JB, Chouaid C, Greillier L, Monnet I, Le Caer H, Falchero L, Corre R, Descourt R, Bota S, Berard H, Schott R, Bizieux A, Fournel P (2014). Randomized open-label non-comparative multicenter phase II trial of sequential erlotinib and docetaxel versus docetaxel alone in patients with non-small-cell lung cancer after failure of first-line chemotherapy: GFPC 10. 02 study. Lung cancer.

[13] Boutsikou E, Kontakiotis T, Zarogoulidis P, Darwiche K, Eleptheriadou E, Porpodis K, Galaktidou G, Sakkas L, Hohenforst-Schmidt W, Tsakiridis K, Karaiskos T, Zarogoulidis K (2013). Docetaxel-carboplatin in combination with erlotinib and/or bevacizumab in patients with non-small cell lung cancer. Onco Targets Ther.

[14] Gatzemeier U, Pluzanska A, Szczesna A, Kaukel E, Roubec J, De Rosa F, Milanowski J, Karnicka-Mlodkowski H, Pesek M, Serwatowski P, Ramlau R, Janaskova T, Vansteenkiste J (2007). Phase III study of erlotinib in combination with cisplatin and gemcitabine in advanced non-small-cell lung cancer: the Tarceva Lung Cancer Investigation Trial. J Clin Oncol.

[15] Giaccone G, Herbst RS, Manegold C, Scagliotti G, Rosell R, Miller V, Natale RB, Schiller JH, Von Pawel J, Pluzanska A, Gatzemeier U, Grous J, Ochs JS (2004). Gefitinib in combination with gemcitabine and cisplatin in advanced non-small-cell lung cancer: a phase III trial—INTACT 1. J Clin Oncol.

[16] Herbst RS, Giaccone G, Schiller JH, Natale RB, Miller V, Manegold C, Scagliotti G, Rosell R, Oliff I, Reeves JA, Wolf MK, Krebs AD, Averbuch SD (2004). Gefitinib in combination with paclitaxel and carboplatin in advanced non-small-cell lung cancer: a phase III trial—INTACT 2. J Clin Oncol.

[17] Herbst RS, Prager D, Hermann R, Fehrenbacher L, Johnson BE, Sandler A, Kris MG, Tran HT, Klein P, Li X, Ramies D, Johnson DH, Miller VA (2005). TRIBUTE: a phase III trial of erlotinib hydrochloride (OSI-774) combined with carboplatin and paclitaxel chemotherapy in advanced non-small-cell lung cancer. J Clin Oncol.

[18] Hirsch FR, Kabbinavar F, Eisen T, Martins R, Schnell FM, Dziadziuszko R, Richardson K, Richardson F, Wacker B, Sternberg DW, Rusk J, Franklin WA, Varella-Garcia M (2011). A randomized, phase II, biomarker-selected study comparing erlotinib to erlotinib intercalated with chemotherapy in first-line therapy for advanced non-small-cell lung cancer. J Clin Oncol.

[19] Janne PA, Wang X, Socinski MA, Crawford J, Stinchcombe TE, Gu L, Capelletti M, Edelman MJ, Villalona-Calero MA, Kratzke R, Vokes EE, Miller VA (2012). Randomized phase II trial of erlotinib alone or with carboplatin and paclitaxel in patients who were never or light former smokers with advanced lung adenocarcinoma: CALGB 30406 trial. J Clin Oncol.

[20] Lee DH, Lee JS, Kim SW, Rodrigues-Pereira J, Han B, Song XQ, Wang J, Kim HK, Sahoo TP, Digumarti R, Wang X, Altug S, Orlando M (2013). Three-arm randomised controlled phase 2 study comparing pemetrexed and erlotinib to either pemetrexed or erlotinib alone as second-line treatment for never-smokers with non-squamous non-small cell lung cancer. Eur J Cancer.

[21] Mok TS, Wu YL, Yu CJ, Zhou C, Chen YM, Zhang L, Ignacio J, Liao M, Srimuninnimit V, Boyer MJ, Chua-Tan M, Sriuranpong V, Sudoyo AW (2009). Randomized, placebo-controlled, phase II study of sequential erlotinib and chemotherapy as first-line treatment for advanced non-small-cell lung cancer. J Clin Oncol.

[22] Soria JC, Wu YL, Nakagawa K, Kim SW, Yang JJ, Ahn MJ, Wang J, Yang JC, Lu Y, Atagi S, Ponce S, Lee DH, Liu Y (2015). Gefitinib plus chemotherapy versus placebo plus chemotherapy in EGFR-mutation-positive non-small-cell lung cancer after progression on first-line gefitinib (IMPRESS): a phase 3 randomised trial. Lancet Oncology.

[23] Yu H, Zhang J, Wu X, Luo Z, Wang H, Sun S, Peng W, Qiao J, Feng Y, Wang J, Chang J (2014). A phase II randomized trial evaluating gefitinib intercalated with pemetrexed/platinum chemotherapy or pemetrexed/platinum chemotherapy alone in unselected patients with advanced non-squamous non-small cell lung cancer. Cancer Biol Ther.

[24] Wu YL, Lee JS, Thongprasert S, Yu CJ, Zhang L, Ladrera G, Srimuninnimit V, Sriuranpong V, Sandoval-Tan J, Zhu Y, Liao M, Zhou C, Pan H (2013). Intercalated combination of chemotherapy and erlotinib for patients with advanced stage non-small-cell lung cancer (FASTACT-2): a randomised, double-blind trial. Lancet Oncology.

[25] Aerts JG, Codrington H, Lankheet NA, Burgers S, Biesma B, Dingemans AM, Vincent AD, Dalesio O, Groen HJ, Smit EF (2013). A randomized phase II study comparing erlotinib versus erlotinib with alternating chemotherapy in relapsed non-small-cell lung cancer patients: the NVALT-10 study. Ann Oncol.

[26] Dittrich C, Papai-Szekely Z, Vinolas N, Sederholm C, Hartmann JT, Behringer D, Kazeem G, Desaiah D, Leschinger MI, von Pawel J (2014). A randomised phase II study of pemetrexed versus pemetrexed+erlotinib as second-line treatment for locally advanced or metastatic non-squamous non-small cell lung cancer. Eur J Cancer.

[27] Rossi A, Chiodini P, Sun JM, O'Brien ME, von Plessen C, Barata F, Park K, Popat S, Bergman B, Parente B, Gallo C, Gridelli C, Perrone F (2014). Six versus fewer planned cycles of first-line platinum-based chemotherapy for non-small-cell lung cancer: a systematic review and meta-analysis of individual patient data. The Lancet Oncology.

[28] Russo A, Franchina T, Ricciardi GR, Picone A, Ferraro G, Zanghi M, Toscano G, Giordano A, Adamo V (2015). A decade of EGFR inhibition in EGFR-mutated non small cell lung cancer (NSCLC): Old successes and future perspectives. Oncotarget.

[29] Li T, Ling YH, Goldman ID, Perez-Soler R (2007). Schedule-dependent cytotoxic synergism of pemetrexed and erlotinib in human non-small cell lung cancer cells. Clin Cancer Res.

[30] Giovannetti E, Lemos C, Tekle C, Smid K, Nannizzi S, Rodriguez JA, Ricciardi S, Danesi R, Giaccone G, Peters GJ (2008). Molecular mechanisms underlying the synergistic interaction of erlotinib, an epidermal growth factor receptor tyrosine kinase inhibitor, with the multitargeted antifolate pemetrexed in non-small-cell lung cancer cells. Mol Pharmacol.

[31] Xu JL, Jin B, Ren ZH, Lou YQ, Zhou ZR, Yang QZ, Han BH (2015). Chemotherapy plus Erlotinib versus Chemotherapy Alone for Treating Advanced Non-Small Cell Lung Cancer: A Meta-Analysis. PloS one.

[32] Ge L, Shi R (2015). Progress of EGFR-TKI and ALK/ROS1 inhibitors in advanced non-small cell lung cancer. Int J Clin Exp Med.

[33] Ha SY, Choi SJ, Cho JH, Choi HJ, Lee J, Jung K, Irwin D, Liu X, Lira ME, Mao M, Kim HK, Choi YS, Shim YM (2015). Lung cancer in never-smoker Asian females is driven by oncogenic mutations, most often involving EGFR. Oncotarget.

[34] Pao W, Miller V, Zakowski M, Doherty J, Politi K, Sarkaria I, Singh B, Heelan R, Rusch V, Fulton L, Mardis E, Kupfer D, Wilson R (2004). EGF receptor gene mutations are common in lung cancers from “never smokers” and are associated with sensitivity of tumors to gefitinib and erlotinib. Proc Natl Acad Sci U S A.

[35] Piperdi B, Ling YH, Perez-Soler R (2007). Schedule-dependent interaction between the proteosome inhibitor bortezomib and the EGFR-TK inhibitor erlotinib in human non-small cell lung cancer cell lines. J Thorac Oncol.

[36] Feld R, Sridhar SS, Shepherd FA, Mackay JA, Evans WK (2006). Use of the epidermal growth factor receptor inhibitors gefitinib and erlotinib in the treatment of non-small cell lung cancer: a systematic review. J Thorac Oncol.

[37] Davies AM, Ho C, Lara PN, Mack P, Gumerlock PH, Gandara DR (2006). Pharmacodynamic separation of epidermal growth factor receptor tyrosine kinase inhibitors and chemotherapy in non-small-cell lung cancer. Clin Lung Cancer.

[38] Cappuzzo F, Ciuleanu T, Stelmakh L, Cicenas S, Szczesna A, Juhasz E, Esteban E, Molinier O, Brugger W, Melezinek I, Klingelschmitt G, Klughammer B, Giaccone G (2010). Erlotinib as maintenance treatment in advanced non-small-cell lung cancer: a multicentre, randomised, placebo-controlled phase 3 study. Lancet Oncology.

[39] Zhang L, Ma S, Song X, Han B, Cheng Y, Huang C, Yang S, Liu X, Liu Y, Lu S, Wang J, Zhang S, Zhou C (2012). Gefitinib versus placebo as maintenance therapy in patients with locally advanced or metastatic non-small-cell lung cancer (INFORM; C-TONG 0804): a multicentre, double-blind randomised phase 3 trial. Lancet Oncology.

[40] OuYang PY, Su Z, Mao YP, Deng W, Xie FY (2013). Combination of EGFR-TKIs and chemotherapy as first-line therapy for advanced NSCLC: a meta-analysis. PloS one.

[41] Moher D, Liberati A, Tetzlaff J, Altman DG (2010). Preferred reporting items for systematic reviews and meta-analyses: the PRISMA statement. Int J Surg.

[42] Tierney JF, Stewart LA, Ghersi D, Burdett S, Sydes MR (2007). Practical methods for incorporating summary time-to-event data into meta-analysis. Trials.

[43] Jadad AR, Moore RA, Carroll D, Jenkinson C, Reynolds DJ, Gavaghan DJ, McQuay HJ (1996). Assessing the quality of reports of randomized clinical trials: is blinding necessary?. Control Clin Trials.

