# Supplementary material for: Efficacy of epidermal growth factor receptor inhibitors in combination with chemotherapy in advanced non-small cell lung cancer: A meta-analysis of randomized controlled trials
Source: Oncotarget. 2016 May 20;7(26):39823–33. doi: 10.18632/oncotarget.9503 (PMC5129973; doi:10.18632/oncotarget.9503)
Supplement: Supplementary file 1 [file oncotarget-07-39823-s001.pdf]

Efficacy of epidermal growth factor receptor inhibitors in combination with chemotherapy in advanced non-small cell lung cancer: A meta-analysis of randomized controlled trials

Supplementary Materials

Study or Subgroup	log[Hazard Ratio]	SE	Weight	Hazard Ratio IV, Random, 95% CI
Auliac 2014	-0.0408	0.1625	11.5%	0.96 [0.70, 1.32]
Dittrich 2014	-0.462	0.182	10.6%	0.63 [0.44, 0.90]
Giaccone 2004	-0.0513	0.0885	15.5%	0.95 [0.80, 1.13]
Herbst 2004	-0.1508	0.082	15.8%	0.86 [0.73, 1.01]
Mok 2009	-0.755	0.1877	10.3%	0.47 [0.33, 0.68]
Soria 2015	-0.1508	0.1393	12.8%	0.86 [0.65, 1.13]
Wu 2013	-0.5621	0.0975	15.0%	0.57 [0.47, 0.69]
Yu 2014	-0.1278	0.2273	8.6%	0.88 [0.56, 1.37]
**Total (95% CI)**			**100.0%**	**0.76 [0.63, 0.91]**

Heterogeneity: Tau² = 0.05; Chi² = 27.79, df = 7 (P = 0.0002); I² = 75%
Test for overall effect: Z = 3.03 (P = 0.002)

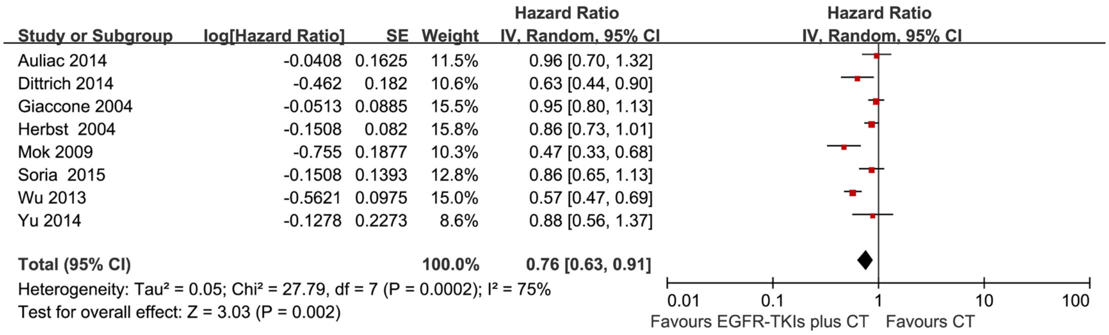

Supplementary Figure S1: Forest plot for PFS comparing EGFR-TKIs plus CT with CT

Study or Subgroup	log[Hazard Ratio]	SE	Weight	Hazard Ratio IV, Random, 95% CI
Aerts 2013	-0.268	0.1448	8.0%	0.76 [0.58, 1.02]
Gatzemeier 2007	-0.0212	0.0641	40.6%	0.98 [0.86, 1.11]
Herbst 2005	-0.0576	0.0625	42.7%	0.94 [0.84, 1.07]
Hirsh 2011	-0.0305	0.219	3.5%	0.97 [0.63, 1.49]
Janne 2012	-0.2107	0.1788	5.2%	0.81 [0.57, 1.15]
**Total (95% CI)**			**100.0%**	**0.94 [0.86, 1.01]**

Heterogeneity: Tau² = 0.00; Chi² = 3.13, df = 4 (P = 0.54); I² = 0%
Test for overall effect: Z = 1.63 (P = 0.10)

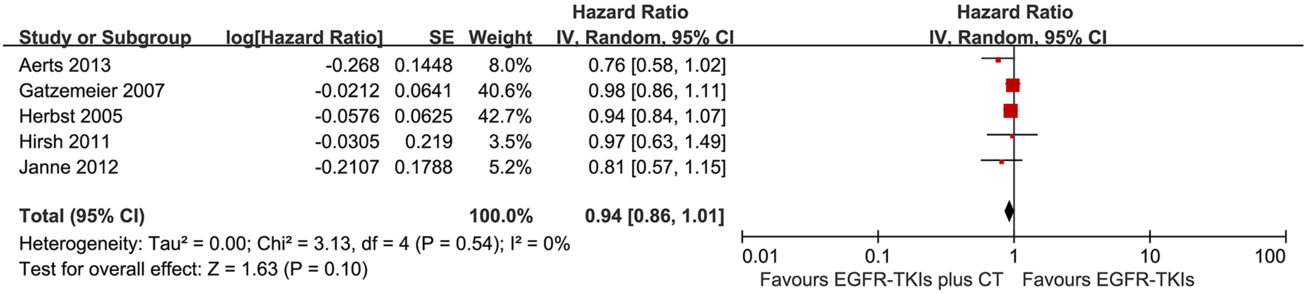

Supplementary Figure S2: Forest plot for PFS comparing EGFR-TKIs plus CT with EGFR-TKIs

Study or Subgroup	log[Hazard Ratio]	SE	Weight	Hazard Ratio IV, Random, 95% CI
Boutsikou2013	-0.2231	0.3831	2.9%	0.80 [0.38, 1.70]
Dittrich 2014	-0.3857	0.1865	10.5%	0.68 [0.47, 0.98]
Giaccone 2004	0.0583	0.0962	26.1%	1.06 [0.88, 1.28]
Herbst 2004	0.01	0.094	26.7%	1.01 [0.84, 1.21]
Mok 2009	0.0862	0.2238	7.7%	1.09 [0.70, 1.69]
Wu 2013	-0.2357	0.1151	21.2%	0.79 [0.63, 0.99]
Yu 2014	-0.1744	0.289	4.9%	0.84 [0.48, 1.48]
**Total (95% CI)**			**100.0%**	**0.92 [0.81, 1.05]**

Heterogeneity: Tau² = 0.01; Chi² = 8.21, df = 6 (P = 0.22); I² = 27%
Test for overall effect: Z = 1.21 (P = 0.23)

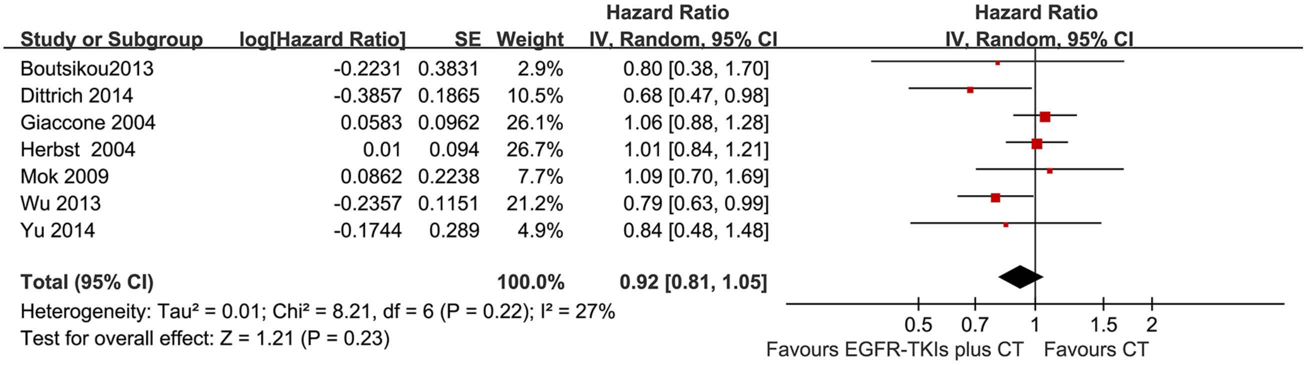

Supplementary Figure S3: Forest plot for OS comparing EGFR-TKIs plus CT with CT

Study or Subgroup	log[Hazard Ratio]	SE	Weight	Hazard Ratio IV, Random, 95% CI
Aerts 2013	-0.4005	0.1562	17.2%	0.67 [0.49, 0.91]
Gatzemeier 2007	0.0583	0.0759	31.9%	1.06 [0.91, 1.23]
Herbst 2005	0	0.077	31.7%	1.00 [0.86, 1.16]
Hirsh 2011	0.27	0.2778	7.4%	1.31 [0.76, 2.26]
Janne 2012	0.1044	0.2069	11.8%	1.11 [0.74, 1.67]
**Total (95% CI)**			**100.0%**	**0.98 [0.83, 1.16]**

Heterogeneity: Tau² = 0.02; Chi² = 8.36, df = 4 (P = 0.08); I² = 52%
Test for overall effect: Z = 0.21 (P = 0.83)

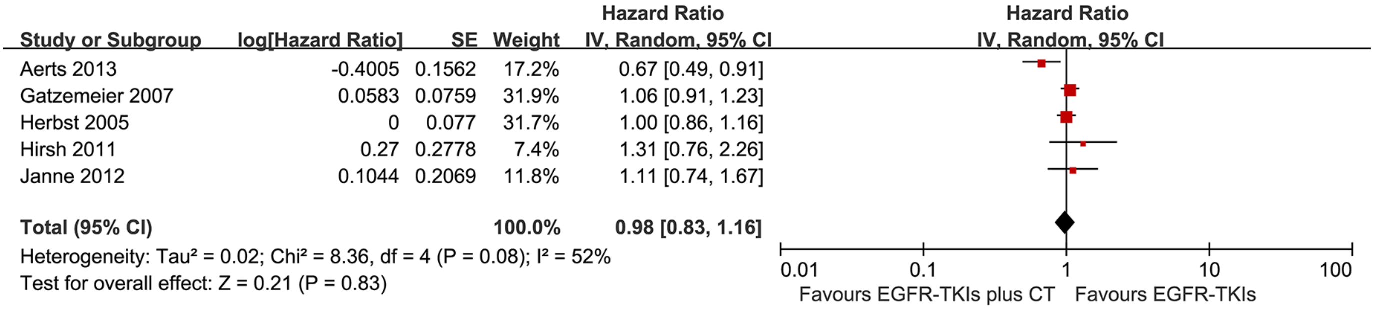

Supplementary Figure S4: Forest plot for OS comparing EGFR-TKIs plus CT with EGFR-TKIs
